# Molecular Cloning, Expression Profiles, Characterization and Function of a Novel Small Heat Shock Protein (*HbsHSP23.8a*) in *Hevea brasiliensis*

**DOI:** 10.3390/plants15213318

**Published:** 2026-10-26

**Authors:** Huina Guo, Jiangshu Chen, Han Yin, Zhihui Xia, Dejun Li

**Affiliations:** 1 State Key Laboratory of Tropical Crop Breeding, Rubber Research Institute, Chinese Academy of Tropical Agricultural Science, Haikou 571101, China; huinaguo2023@126.com (H.G.); cjshu1627@sina.com (J.C.); 2 Sanya National Center of Technology Innovation for Saline-Alkali Tolerant Rice, School of Breeding and Multiplication (Sanya Institute of Breeding and Multiplication), Hainan University, Sanya 572025, China; 18360574102@163.com; 3 Sanya Research Institute, Chinese Academy of Tropical Agricultural Sciences, Sanya 572025, China

**Keywords:** small heat shock protein (sHSP), salinity stress, rubber tree, abiotic stress, *HbsHSP23.8a*

## Abstract

Small heat shock proteins (sHSPs) are involved in a wide range of abiotic stresses, yet the functional roles of sHSP family genes in rubber tree salt tolerance remain largely uncharacterized. In this study, *HbsHSP23.8a*, a novel sHSP gene from rubber tree, was cloned and analyzed in detail. HbsHSP23.8a consists of a targeting peptide, an N-terminal domain, an α-crystallin domain (ACD), and C-terminal extensions. Additionally, putative dimer interface and conserved amino acid sequences were identified in the ACD and N-terminal domain of HbsHSP23.8a, respectively. *HbsHSP23.8a* transcripts were detected in different tissues and developmental stages of leaves, indicating that *HbsHSP23.8a* was associated with rubber tree growth and development. Besides tapping panel dryness (TPD), *HbsHSP23.8a* was also involved in low-temperature, high-salt, methyl jasmonate (MeJA), and ethephon (ET) responses. Under salt stress, overexpression of *HbsHSP23.8a* in transgenic tobacco plants exhibited greater plant height and fresh weight than wild-type (WT) plants. In addition, tobacco plants overexpressing *HbsHSP23.8a* increased the activities of antioxidant enzymes and decreased hydrogen peroxide (H_2_O_2_) accumulation and chlorophyll degradation under salt stress compared with the WT plants. Collectively, our results suggest that *HbsHSP23.8a* is related to rubber tree growth, TPD, leaf development, stress and hormone responses. Furthermore, heterologous expression of *HbsHSP23.8a* is associated with enhanced salt tolerance in transgenic tobacco, which may be attributed to elevated antioxidant enzyme activities, reduced cellular H_2_O_2_ accumulation, and alleviated chlorophyll degradation.

## 1. Introduction

Salt stress arises from excessive accumulation of soluble salts in soil. Soil salinity is often worsened by human activities (e.g., irrigation with river water and overgrazing) and environmental factors including arid climates, rising sea levels, seawater intrusion, and proximity to saltwater bodies [1,2]. According to statistics released by the Food and Agriculture Organization (FAO) in October 2021, more than 833 million hectares of farmland have been impacted by salinization. In addition, ~1.5 million hectares of land became unfit for cultivation because of excessive soil salinity [3]. The net changes in soil salinity and the total extent of salt-affected soils exhibit substantial geographic variability, but the continents with the most extensive salt-affected soils are Asia (notably China, Kazakhstan, and Iran), Africa, and Australia. In terms of the areal coverage of salt-affected lands, China (211.74 Mha) is the largest country, followed by Australia (131.40 Mha), Kazakhstan (93.31 Mha), and Iran (88.33 Mha) [4]. Salinized soils contain abundant soluble ions, mainly including chloride, sulfate, sodium, potassium, magnesium, and calcium ions [5,6,7]. Salt stress elicits a suite of primary physiological perturbations (osmotic stress, reduced nutrient acquisition, and suppressed plant growth) as well as more intricate secondary imbalances, among which the excessive generation of reactive oxygen species (ROS) and free radicals poses a critical threat by inducing oxidative damage to proteins, membrane lipids, carbohydrates, and DNA structures [8,9,10]. In addition to threatening plant growth and global agricultural productivity, salt stress also imposes adverse impacts on global ecology and resources [11].

To effectively cope with salt stress, plants initiate a series of sophisticated adaptive mechanisms to alleviate salt-induced damage and maintain intracellular homeostasis. Small heat shock proteins (sHSPs) serve as crucial functional molecules for plants to defend against salt stress [12]. sHSPs are low-molecular-weight polypeptides with a molecular mass of 12–25 kDa. They are categorized as HSP20/α-crystallin proteins (PFAM family: HSP20/α-crystallin, accession number PF00011), and the α-crystallin domain (ACD) is the conserved signature domain of the sHSPs [13]. The ACD, approximately 90 amino acids in size, forms a compact seven-stranded β-sandwich structure that constitutes the structural core of all sHSPs [14]. In plants, sHSPs are usually participated in various stress responses and are hypothesized to function as molecular chaperones, safeguarding other proteins against stress-induced damage. Originally, sHSPs were recognized as heat stress-induced proteins [15]. However, more and more subsequent studies have shown that sHSPs also participate in responses to a wide range of abiotic stresses, including drought, salinity, cold, and heavy metals [16,17,18,19,20,21,22].

The rubber tree (*Hevea brasiliensis*) is an economically indispensable tropical crop worldwide. As the core product of rubber trees, natural rubber possesses superior and unique physicochemical properties that cannot be fully substituted by synthetic rubber materials, making it an indispensable industrial raw material [23]. Beyond extensive industrial value, natural rubber also holds significant status as a strategic commodity. Salinity stress severely impairs rubber tree growth by significantly reducing the root-to-shoot ratio and seed vigor [24]. Yang et al. (2020) demonstrated that salinity stress might lead to chlorophyll degradation and H_2_O_2_ accumulation in rubber tree seedlings [25]. With the continuous expansion of salinized land areas, rubber trees are facing increasingly severe threats from salt stress. Therefore, improving the salt tolerance of rubber trees is of crucial significance for alleviating the adverse impacts of salinity on their growth and latex productivity.

Two strategies are commonly adopted to enhance the salt tolerance of rubber trees. The first strategy involves the application of plant growth regulators, which can effectively improve the plants’ adaptability to salt stress. For instance, Yang et al. (2020) demonstrated that melatonin enhances the salt stress tolerance of rubber tree seedlings by counteracting the cellular accumulation of H_2_O_2_ [25]. The second strategy focuses on breeding and selection of salt-tolerant rubber tree varieties or clones. In this regard, identifying salt tolerance-associated genes and elucidating their functional mechanisms underlying salt stress tolerance are of great significance. These efforts can not only lay solid theoretical foundations for developing such salt-tolerant germplasm but also provide candidate target genes for improving salt tolerance in rubber tree by transgenic or gene-editing methods. Nevertheless, functional characterization of salt-tolerance genes, especially sHSP family members, has not yet been reported in rubber tree. In this study, a novel sHSP gene, named as *HbsHSP23.8a*, was cloned from rubber tree. We systematically performed bioinformatic characterization, expression pattern, and functional validation of *HbsHSP23.8a*. Consistent with *HbsHSP23.8a* up-regulated by salt stress, heterologous expression of *HbsHSP23.8a* in tobacco plants exhibited greater plant growth, increased antioxidant enzyme activities, reduced H_2_O_2_ accumulation, and alleviated chlorophyll degradation than WT plants under salt stress, suggesting that overexpression of *HbsHSP23.8a* is associated with enhanced salt tolerance in tobacco plants.

## 2. Results

### 2.1. Sequence Analyses of HbsHSP23.8a

**Figure 1:**
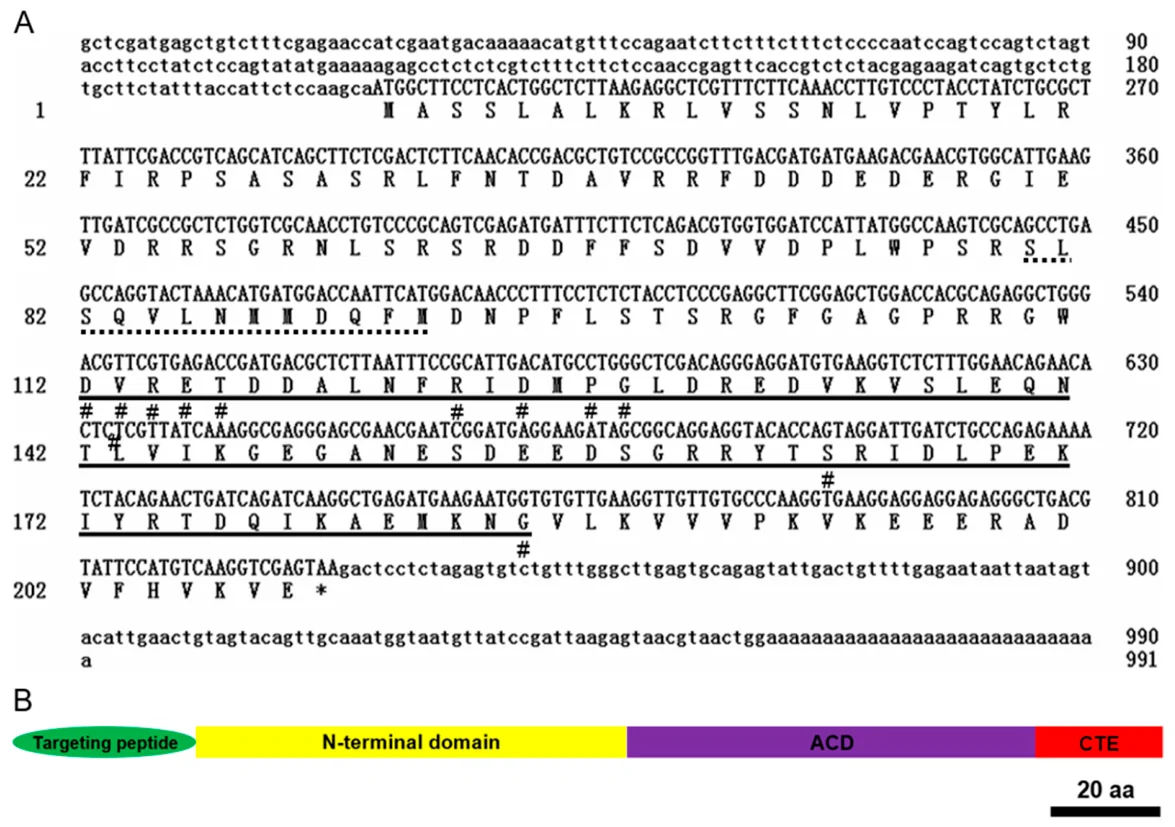
The nucleotide, deduced amino acid sequences and characterization of HbsHSP23.8a. (**A**) The nucleotide, deduced amino acid sequences of HbsHSP23.8a. Amino acids with #, dotted and solid lines represent putative dimer interface (polypeptide binding site), conserved amino acid sequences (SLSQVLNMMDQFM), and ACD, respectively. The regions of ORF and UTRs are block capitals and lowercase letters, respectively, and the termination codon is marked with the asterisk. (**B**) The diagrammatic representation of main domain in HbsHSP23.8a. Green, yellow, pink, and red regions separately present targeting peptide, N-terminal domain, ACD, and C-terminal extensions (CTE).

By analyzing transcriptome data of rubber tree seedlings with salt treatment, we identified one assembled sequence (c40812_g2) with significant homology to plant sHSP [25]. The aforementioned sequence contained one complete open reading frame (ORF) encoding a typical sHSP protein, which was named *HbsHSP23.8a* in this study. According to the c40812_g2 sequence, we designed the specific primers to amplify the 3′- and 5′-UTRs of *HbsHSP23.8a*. The sequences of 3′- and 5′-UTRs from *HbsHSP23.8a* were obtained by sequencing their PCR products inserted into the pNC-AEnTopo vector. After aligning and assembling the sequences including 3′-UTR, the c40812_g2, and 5′-UTRs, we gained *HbsHSP23.8a* full-length cDNA and validated it by sequencing its PCR products. As shown in Figure 1A, the full-length cDNA of *HbsHSP23.8a* is 991 bp in length and contains a complete ORF. The lengths of 5′- and 3′-UTRs are separately 206 bp and 158 bp. The predicted ORF within *HbsHSP23.8a* encodes a polypeptide of 208 amino acids, with a theoretical isoelectric point (pI) of 5.20 and a molecular weight of 23.83 kDa.

By searching for NCBI conserved domains within HbsHSP23.8a protein, we found that HbsHSP23.8a belongs to the Hsp20/alpha crystallin family protein, which functions as an ATP-independent chaperone that prevents aggregation and protects against cell stress, induced by heat, osmotic, or acid shock. Besides ACD, HbsHSP23.8a also contains a targeting peptide, an N-terminal domain, and C-terminal extensions (Figure 1B). Additionally, putative dimer interface (polypeptide binding site) and conserved amino acid sequences (SLSQVLNMMDQFM) are separately located in the ACD and N-terminal domain of HbsHSP23.8a (Figure 1B). Collectively, the aforementioned conserved domain and structural features indicate that HbsHSP23.8a likely exerts typical sHSP functions in rubber tree.

### 2.2. Expression Profiles of HbsHSP23.8a

**Figure 2:**
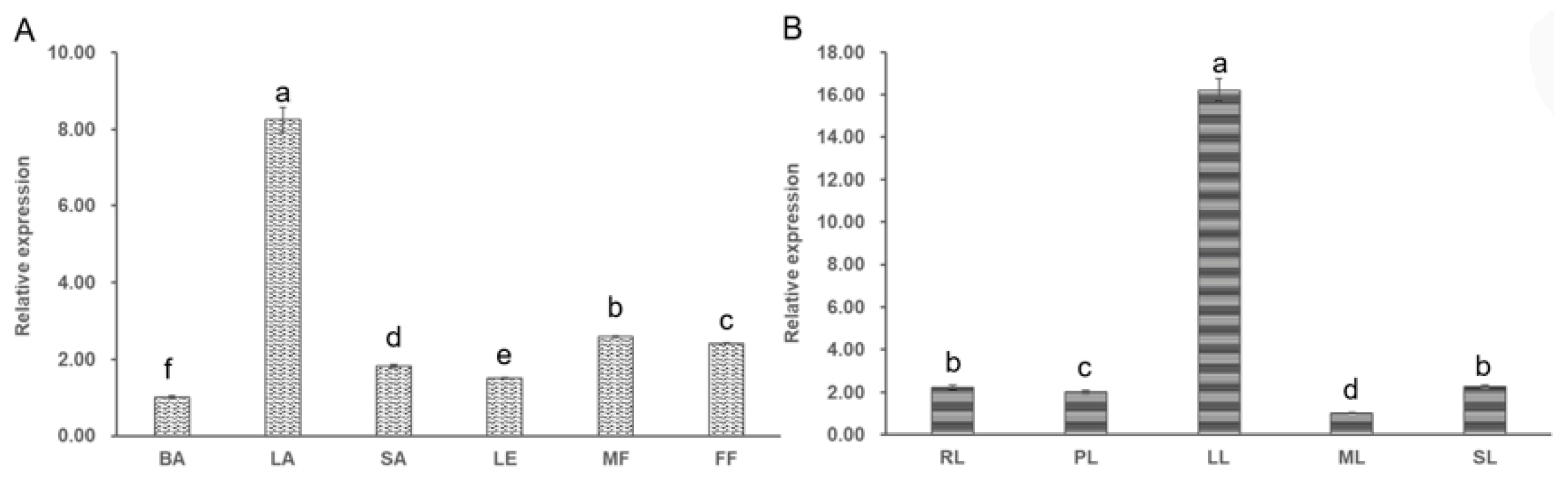
Expression patterns of *HbsHSP23.8a* in different tissues and leaf developmental stages of rubber trees. (**A**) Expression patterns of *HbsHSP23.8a* in six rubber tree tissues, with bark used as the reference for relative expression quantification. BA, LA, SA, LE, MF, and FF separately indicate barks, latex, stem apexes, leaves, male flowers, and female flowers. (**B**) Expression profiles of *HbsHSP23.8a* in the five leaf developmental stages, with mature leaf serving as the reference for relative expression. Red, pale-green young, light young, mature, and senescence leaves are represented with RL, PL, LL, ML, and SL represent, respectively. All values are represented the mean ± standard deviation (SD) of three biological replicates. For each panel, differences among groups were analyzed by one-way ANOVA followed by Duncan’s multiple range test (DMRT). Different lowercase letters above the bars indicate significant differences among groups at *p* < 0.05.

**Figure 3:**
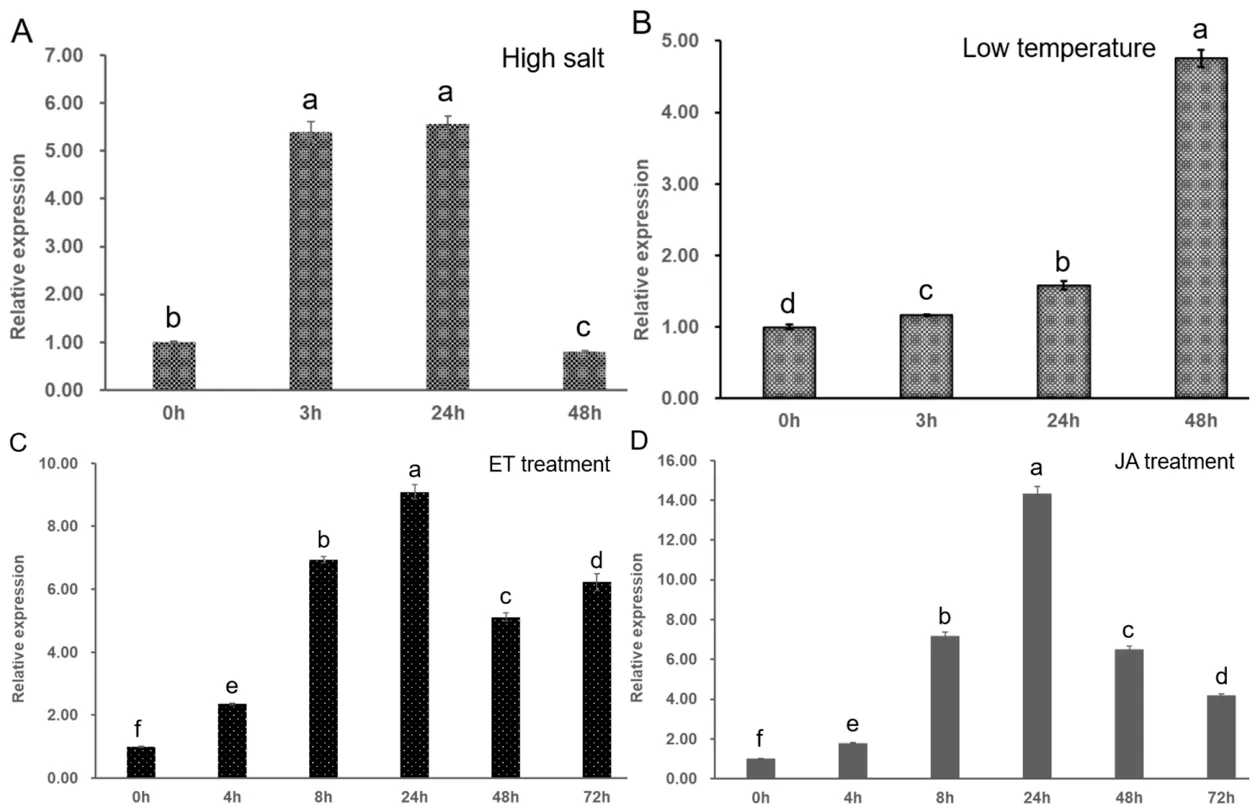
Expression patterns of *HbsHSP23.8a* under high salt, low temperature, ET, and JA treatments. (**A**) Expression profiles of *HbsHSP23.8a* in leaves under high salt treatment; (**B**) expression patterns of *HbsHSP23.8a* in leaves under low-temperature treatment; (**C**) expression profiles of *HbsHSP23.8a* in latex under ET treatment; (**D**) expression profiles of *HbsHSP23.8a* in latex under JA treatment. Values represent the mean ± SD of three biological replicates. Each treatment series was analyzed individually using one-way ANOVA followed by DMRT. Different lowercase letters above the bars denote significant differences among time points at *p* < 0.05.

**Figure 4:**
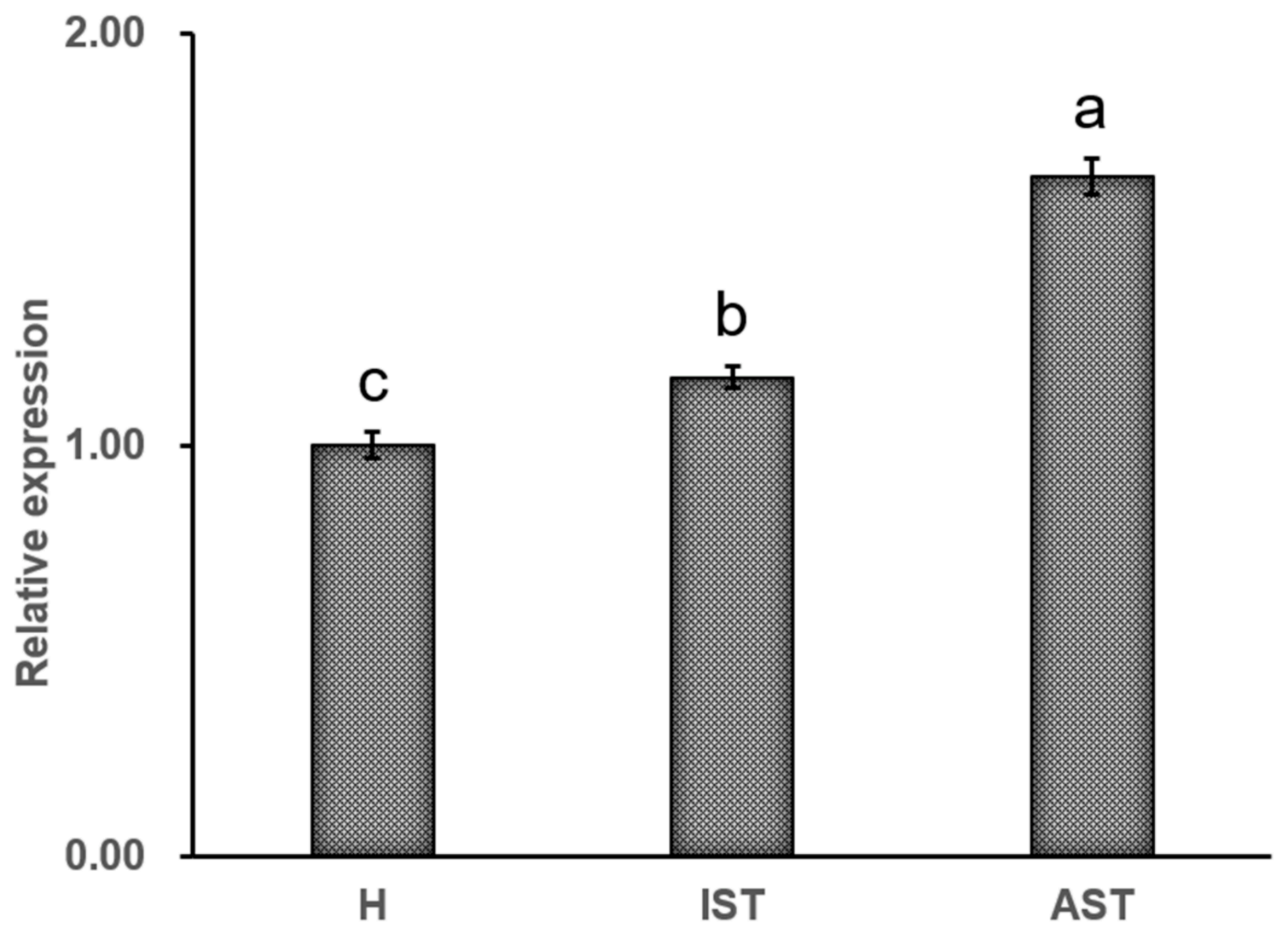
Expression profiles of *HbsHSP23.8a* in barks from TPD-affected and healthy rubber trees. IST, AST, and H represent bark samples harvested from rubber trees in the initial TPD stage, advanced TPD stage, and healthy rubber trees, respectively. Values represent the mean ± SD of three biological replicates. Differences among H, IST, and AST were analyzed using one-way ANOVA followed by DMRT. Different lowercase letters above the bars denote significant differences among the three groups at *p* < 0.05.

To explore the potential biological functions of *HbsHSP23.8a*, we further analyzed expression patterns of *HbsHSP23.8a* by the qRT-PCR method. As shown in Figure 2A, *HbsHSP23.8a* highly expressed in latex, followed by male flowers, female flowers, stem apexes, leaves, and barks. In terms of leaf developmental stages, *HbsHSP23.8a* expression in the leaves in the pale-green young stage was obviously higher than in those in the other four stages including red, light-young, mature and senescence (Figure 2B). Besides different tissues and leaf development stages, *HbsHSP23.8a* expressions were also investigated with four different treatments. Under high salt stress, *HbsHSP23.8a* was rapidly and strongly induced, with expression levels increasing more than 5-fold at 3 h and remaining elevated at 24 h, before declining to near-baseline levels by 48 h (Figure 3A). Under low temperature treatment, *HbsHSP23.8a* exhibited a gradual and sustained up-regulation, with transcript levels rising progressively from 0 h to 48 h and reaching a maximum (~5-fold increase) at 48 h (Figure 3B). ET treatment triggered a time-dependent induction of *HbsHSP23.8a*, with expression increasing steadily from 0 h, peaking at 24 h (~9-fold above the control), and subsequently decreasing at 48 h and 72 h, although it remained significantly higher than the initial level (Figure 3C). Similarly, JA treatment markedly activated *HbsHSP23.8a* expression; the transcript level rose sharply after 8 h, reached its highest point at 24 h (~14-fold induction), and then declined gradually at 48 h and 72 h (Figure 3D). Moreover, *HbsHSP23.8a* expression in barks from the tapping panel dryness (TPD)-affected tree was obviously higher than that from the healthy one, suggesting that *HbsHSP23.8a* was likely associated with TPD occurrence and development (Figure 4). Taken together, the results from expression analyses implied that *HbsHSP23.8a* was a multi-functional gene that participates in TPD, stress, ET and MeJA responses in rubber trees.

### 2.3. HbsHSP23.8a Overexpression in Tobacco Showed Greater Growth Vigor Under Salt Stress

**Figure 5:**
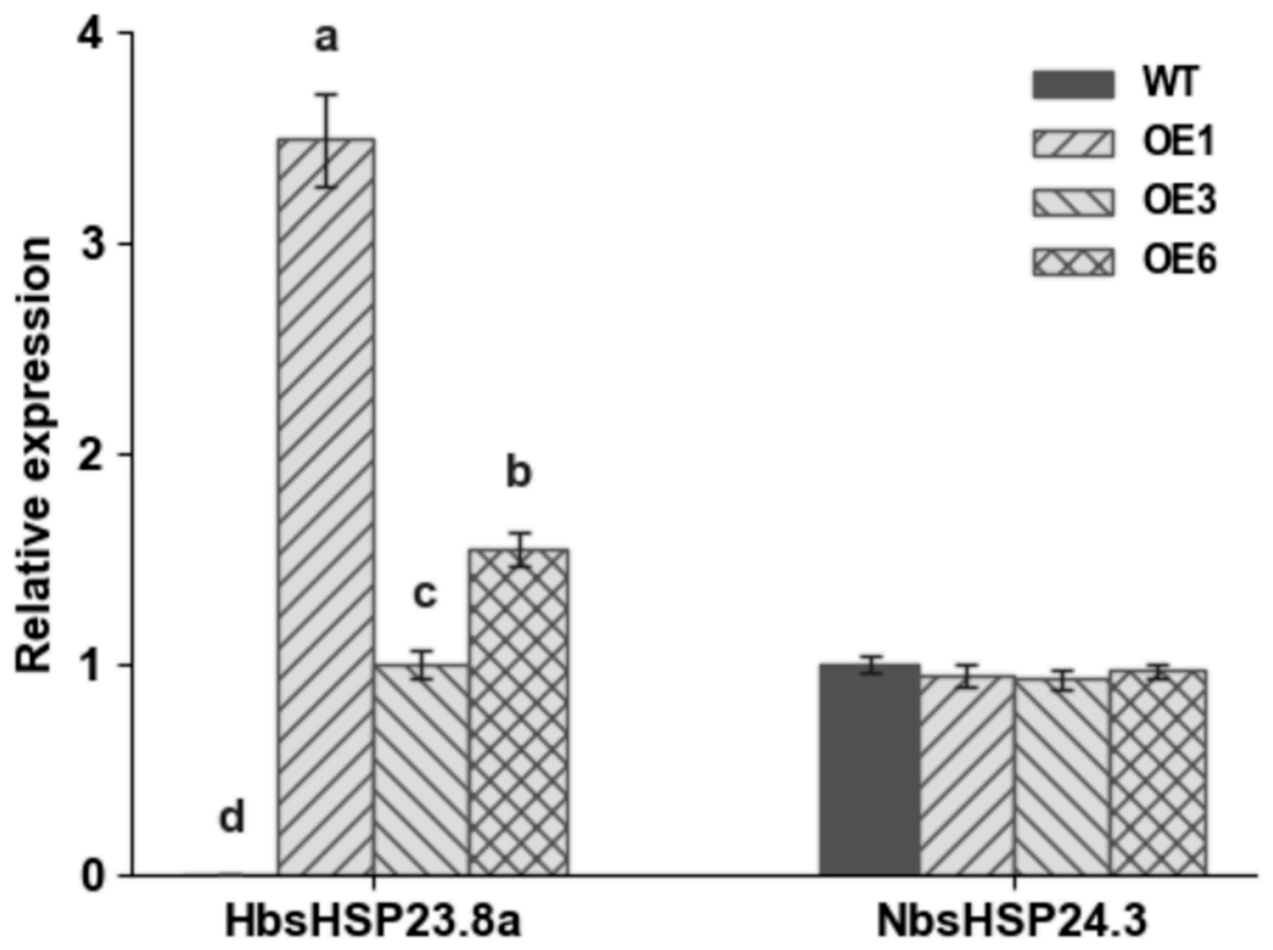
qRT-PCR analysis of *HbsHSP23.8a* and *NbsHSP24.3* expressions in the WT and transgenic tobacco lines overexpressing *HbsHSP23.8a*. Values represent the mean ± SD of three biological replicates. For each gene, differences among the WT and transgenic lines were analyzed individually using one-way ANOVA followed by DMRT. Different lowercase letters above the bars, where present, denote significant differences among genotypes at *p* < 0.05.

To further elucidate the biological function of *HbsHSP23.8a*, we performed *Agrobacterium*-mediated leaf disk transformation to generate transgenic tobacco plants overexpressing *HbsHSP23.8a* under the control of the CaMV35S promoter. A total of eight independent *HbsHSP23.8a*-transgenic lines (T_0_ generation) were obtained via hygromycin selection and further confirmed by PCR amplification with CaMV35S forward plus gene-specific reverse primer pairs. Of the aforementioned eight lines, three lines (OE1, OE3 and OE6) exhibited an approximate 3:1 hygromycin resistance segregation ratio in the T_1_ generation, implying that the three lines may insert a single copy of *HbsHSP23.8a*. Accordingly, the homozygous T_3_ seeds of the three transgenic lines were selected for further experiments. As shown in Figure 5, the expression levels of *HbsHSP23.8a* were significantly different among the three lines in T_3_ generation, whereas no *HbsHSP23.8a* expression was detected in the WT plants. Additionally, the endogenous expression of *NbsHSP24.3* showed no obvious differences between WT and the three *HbsHSP23.8a*-overexpressing lines, indicating no compensatory expression alteration of this homologous gene in tobacco plants.

**Figure 6:**
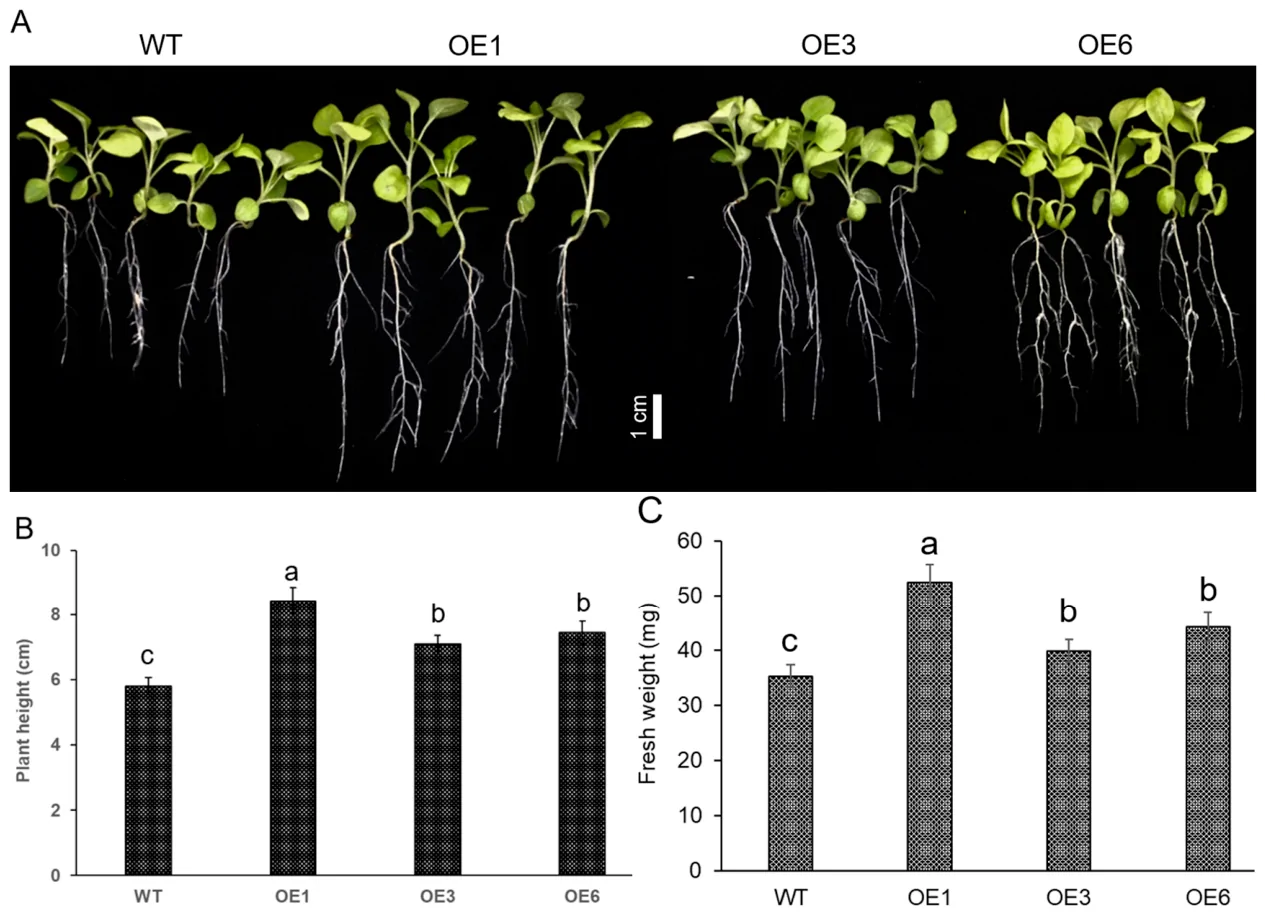
Seedling phenotypes, plant height, and fresh weight from the WT and transgenic tobacco lines overexpressing *HbsHSP23.8a* under salinity stress. (**A**) Phenotypes of seedlings grown on solid MS medium supplemented with 180 mM NaCl for ten days. (**B**) Plant height. (**C**) Plant fresh weight. Values in panels (**B**,**C**) are presented as the mean ± SD of three independent biological replicates. Plant height and fresh weight were analyzed individually using one-way ANOVA followed by DMRT. Different lowercase letters above the bars denote significant differences among genotypes at *p* < 0.05.

Under normal growth conditions, the WT and three *HbsHSP23.8a*-transgenic seedlings exhibited no visible phenotypic differences. Given that *HbsHSP23.8a* was significantly induced by salt treatment, we further evaluated the growth responses of the WT and *HbsHSP23.8a*-transgenic seedlings under 180 mM NaCl treatment. As shown in Figure 6A, a dramatic difference in plant mean height and fresh weight was observed between the transgenic lines and WT seedlings under salt stress. The average plant height and fresh weight of the WT plants were approximately 5.8 cm and 35.3 mg, respectively (Figure 6B,C). In contrast, the three transgenic lines showed greater growth vigor under salt stress, with mean plant heights ranging from 7.1 to 8.4 cm and fresh weights ranging from 39.9 to 52.4 mg (Figure 6B,C). Statistical analysis confirmed that both average plant height and fresh weight of the three transgenic lines were significantly higher than those of the WT under salt stress (Figure 6). Moreover, the expression levels of *HbsHSP23.8a* (rather than endogenous *NbsHSP24.3*) in the three lines were associated with their corresponding plant heights and fresh weights (Figure 5). Collectively, these results suggested that *HbsHSP23.8a* overexpression in tobacco plants showed greater plant height and fresh weight than WT plants under salt stress.

### 2.4. HbsHSP23.8a Is Associated with Enhances Salt Tolerance, Probably by Elevating Antioxidant Enzyme Activities and Reducing H_2_O_2_ Content and Chlorophyll Degradation

**Figure 7:**
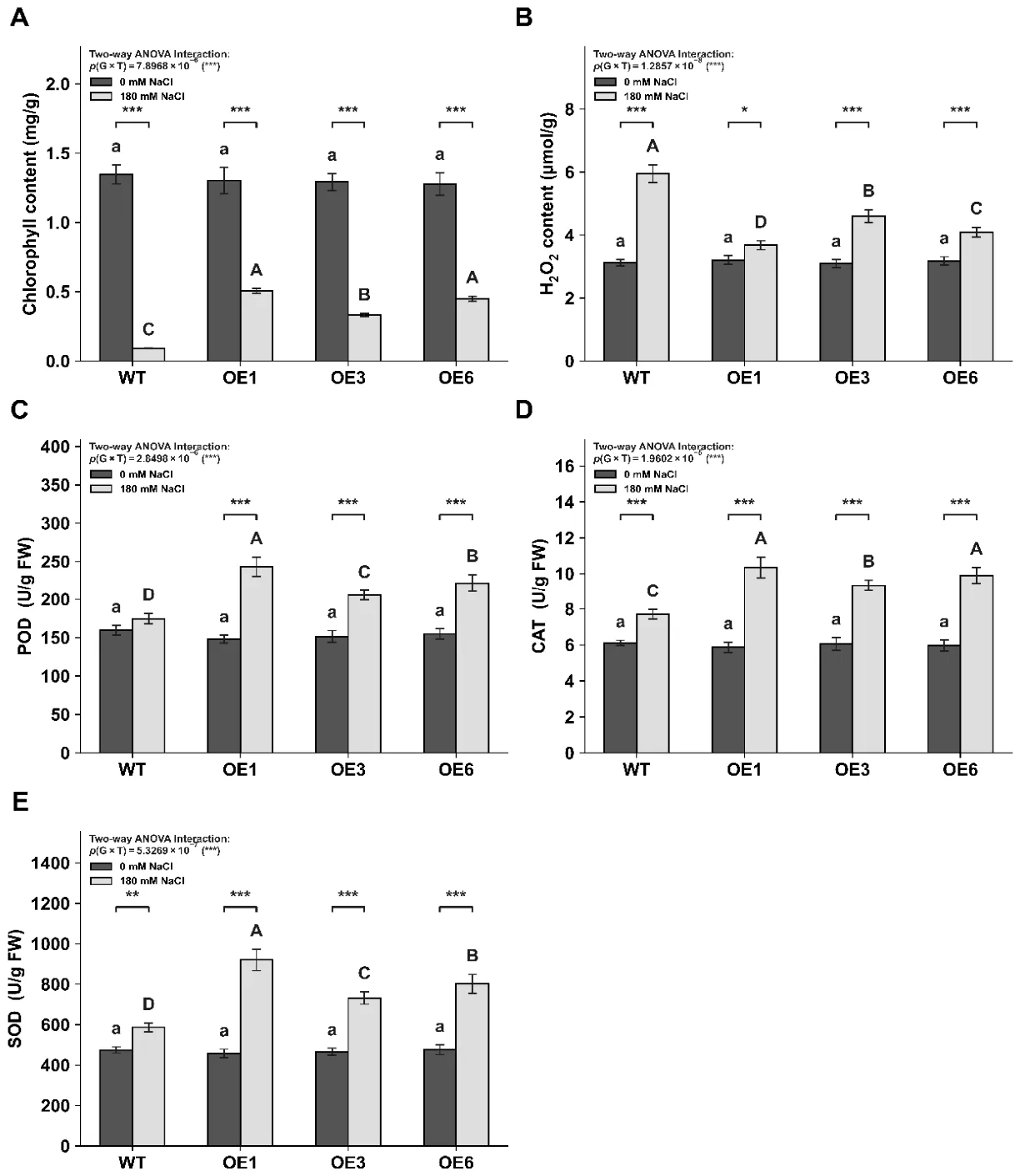
Physiological responses of WT and three *HbsHSP23.8a*-overexpressing tobacco lines under 0 and 180 mM NaCl treatments. Panels (**A**–**E**) represent leaf chlorophyll content, H_2_O_2_ content, POD activity, CAT activity, and SOD activity, respectively. Data are shown as mean ± SD of three independent biological replicates. Two-way ANOVA was applied for each physiological index to assess the main effects of genotype, salt treatment, and their genotype × salt treatment interaction. The *p*-value of the genotype × salt treatment interaction is displayed at the upper-left corner of each panel; significance levels are indicated as *p* < 0.05 (*), *p* < 0.01 (**), and *p* < 0.001 (***). A significant interaction effect triggered simple-effect post hoc comparisons. DMRT, using the pooled residual mean square error (MSE) derived from the two-way ANOVA model, was used to compare genotypes within each salt concentration. Lowercase and uppercase letters denote genotype groupings under 0 mM and 180 mM NaCl, respectively; values with different letters differ significantly at *p* < 0.05. For each genotype, two-sided *t*-tests with Sidak multiple-comparison correction (using the same pooled residual MSE) were used to identify differences between 0 and 180 mM NaCl treatments. Asterisks above brackets mark significant differences between the two salt treatments within a given genotype based on Sidak-adjusted *p* values (*p* < 0.05 (*), *p* < 0.01 (**), and *p* < 0.001 (***)).

Besides growth inhibition, salt stress usually induces excessive H_2_O_2_ accumulation. To investigate the underlying physiological responses, we measured the activities of catalase (CAT), superoxide dismutase (SOD), and peroxidase (POD), as well as H_2_O_2_ and chlorophyll contents in WT and transgenic plants. As shown in Figure 7, two-way ANOVA revealed highly significant genotype × salt treatment interactions for all five physiological indicators (e.g., *P* = 7.8968 × 10^−6^ for chlorophyll content, and *P* = 1.2857 × 10^−8^ for H_2_O_2_ content). Under normal growth conditions, no obvious differences in the H_2_O_2_ and chlorophyll contents were observed between the transgenic plants and WT plants. With salt treatment, the chlorophyll contents in the three transgenic plants were obviously higher than the WT (Figure 7A). Conversely, the transgenic plants exhibited significantly lower H_2_O_2_ content compared with the WT (Figure 7B). Additionally, the corresponding H_2_O_2_ and chlorophyll contents in the WT and transgenic lines indicated significant difference between 0 mM and 180 mM NaCl treatments (Figure 7A,B). We further determined the activities of major ROS-scavenging enzymes, including SOD, CAT, and POD. Under normal growth conditions, only minor variations in the activities of the three enzymes existed between the WT and three transgenic lines. However, salt stress induced substantially higher SOD, POD and CAT activities in transgenic lines compared with the WT (Figure 7C–E), which may account for the altered H_2_O_2_ levels across genotypes. Significant differences in SOD, POD and CAT activities between 0 mM and 180 mM NaCl were also detected in both WT and transgenic plants, with the sole exception of POD activity in the WT (Figure 7C–E). Across the three transgenic lines, the transcript level of *HbsHSP23.8a*, rather than the endogenous *NbsHSP24.3*, was positively correlated with chlorophyll content, CAT, SOD and POD activities, and inversely correlated with H_2_O_2_ contents (Figure 5 and Figure 7). Consistent with these physiological observations, *HbsHSP23.8a* overexpression in tobacco plants showed greater growth vigor than the WT plants under salt stress (Figure 6). Collectively, these results indicate that heterologous expression of *HbsHSP23.8a* is associated with enhanced salt tolerance in tobacco, an effect associated with elevated antioxidant enzyme activities, decreased H_2_O_2_ accumulation, and reduced chlorophyll degradation under salt stress.

## 3. Discussion

sHSPs are ubiquitous molecular chaperones in plants and play important roles in responses to diverse abiotic stresses [16,17,18,19,20,21,22]. In recent years, a growing number of sHSP homologs have been identified and functionally characterized in various plant species [20,26,27,28,29,30,31]. In rubber tree, Ji et al. (2021) identified 12 sHSP family members and further investigated their expression profiles and characteristics in details [32]. Apart from sHSPs, the HSP90 family also participates in rubber tree stress adaptation. Liu et al. (2022) identified 7 *HbHSP90* genes in rubber tree and revealed their differential responses to diverse abiotic and biotic stresses, with *HbHSP90.1* serving as a key stress-responsive member [33]. HbHSP90.1 interacted with HbSGT1b and the aforementioned two proteins were located in the nucleus [33]. Liu et al. (2025) further demonstrated that HbHSP90.3 also interacted with HbSGT1b and fulfilled an essential function in regulating rubber tree tolerance to water deficit [34].

In the present study, a novel sHSP gene, *HbsHSP23.8a*, was identified and cloned from rubber trees. Plant sHSPs are classified into 11 conserved subfamilies in angiosperms according to their amino acid sequence homology, conserved motifs, and subcellular targeting characteristics [14]. Sequence alignment and bioinformatics prediction indicated that *HbsHSP23.8a* probably belongs to the mitochondrial/chloroplast dual-targeted (MTI/CP) sHSP subfamily. Further experimental verification of subcellular localization will be conducted in our subsequent research to confirm its exact cellular distribution. Similar to typical plant MTI/CP sHSPs, HbsHSP23.8a also possesses four conversed structures including targeting peptide, N-terminal domain, ACD, and C-terminal extensions. Moreover, putative dimer interface and the conserved amino acid sequences were separately identified in their ACD and N-terminal domains (Figure 1). The aforementioned conserved domain and structure characteristics strongly supported that *HbsHSP23.8a* might perform the biological role of sHSP protein in rubber tree. Consistent with its conserved structural properties, the expressions of *HbsHSP23.8a* were regulated by abiotic stresses (high salt and low temperature), hormones (ET and MeJA), and TPD occurrence and development in rubber tree. Notably, *HbsHSP23.8a* showed a gradual and continuous increase in transcript abundance only under low temperature stress. Rubber trees are typical tropical species with extreme sensitivity to cold stress, which may account for the relative simplicity of their stress-response pathways. By contrast, the expression patterns of *HbsHSP23.8a* were relatively dynamic and complex under high salt, ET, and JA treatments; *HbsHSP23.8a* showed transient strong induction under high salt stress, and time-dependent induction followed by decline upon ET and JA treatments. High salt, ET, and JA treatments are implicated in various physiological processes of rubber trees such as growth regulation, latex metabolism, and defense responses, etc., which consequently gives rise to the dynamic and complex expression patterns of *HbsHSP23.8a*. Similar to our results, most of the 12 *HbsHSPs* and *HbHSP90.3* exhibited dynamic and complex expression patterns with ET treatments [32,34]. Additionally, *HbHSP90.1* and *HbHSP90.3* showed diverse and intricate expression profiles with JA treatments [33,34]. in terms of expression patterns during TPD occurrence and development, *HbsHSP23.8a* exhibited a similar expression trend to *HbsHSP16.2* among three TPD stages including H, IST, and AST [32]. These results suggest that *HbsHSP23.8a* may serve as a multifunctional gene that integrates stress signals, phytohormone signaling, and TPD regulatory pathways. The great majority of the 12 *HbsHSPs* differently expressed in five tissues and leaf at four developmental stages [32]. Intriguingly, the expression level of *HbsHSP23.8a* in latex was obviously higher than in the other five tissues. Among five developmental stages of leaf, *HbsHSP23.8a* expression in the leaves at the pale-green young stage was significantly higher than at the other four stages. Such unique expression characteristics imply that *HbsHSP23.8a* may exert predominant biological functions in latex and the leaves at pale-green young stage.

Salinity has become a major environmental constraint on global crop productivity [35,36]. Salt stress can interrupt plant osmotic balance and water relations, thereby triggering cellular damage and impairing normal metabolic processes [3,37,38]. Furthermore, salt stress can lead to excessive production and accumulation of ROS, which contributes to chlorophyll degradation and ultimately reduces photosynthetic efficiency and cell injury in plants [10,39,40]. Plant sHSPs have been reported to carry out both positive and negative regulatory effects under salt stress. Two studies reported that the overexpression of *AsHSP17* or *ClHSP22.8* in *Arabidopsis* negatively regulated plant responses to salt stress [12,17]. By contrast, numerous studies have overexpressed different sHSPs in homologous and heterologous *E. coli*, yeasts, and plant systems [21,41,42,43,44,45,46,47,48,49], and their results indicated that sHSPs had positive effects on salt stress tolerance. Being consistent with the results mentioned above, the transgenic plants showed better phenotypic morphology than the WT lines under salt stress, such as greater plant height and fresh weight. Moreover, overexpression of *HbsHSP23.8a* increases the activities of antioxidant enzymes including CAT, SOD, and POD and decreases H_2_O_2_ accumulation and chlorophyll degradation compared with the WT under salt stress. Interestingly, SOD, POD, and CAT activities showed only minor variations between the WT and three transgenic lines under normal growth conditions. However, significant differences in the activities of the three enzymes were detected between 0 mM and 180 mM NaCl treatments in both WT and transgenic plants, except for POD activity in the WT. In *Arabidopsis*, SOD and POD activities were significantly reduced, while CAT activity increased markedly under salt stress [49]. In *Lycium ruthenicum*, SOD and CAT activities increased sharply with rising salt concentration, whereas POD activity decreased under nearly all NaCl treatments across all tissues relative to the control [50]. Therefore, the regulatory function of POD in salt stress response remains unclear. All the aforementioned results suggested that *HbsHSP23.8a* overexpression is associated with enhanced growth and tolerance to salt stress in tobacco plants.

TPD is a complex physiological disorder triggered by overexploitation (overstimulation with ethylene and/or excessive tapping) [51,52,53]. Multiple key pathways participate in TPD occurrence and development in rubber trees, including ROS production and scavenging, the ubiquitin–proteasome pathway (UPP), programmed cell death (PCD), JA signaling, and rubber biosynthesis [54,55,56,57,58,59]. Li et al. proposed that overexploitation induces excessive ROS generation, disrupting the equilibrium between ROS production and scavenging and leading to ROS accumulation. As cytotoxic molecules, excess ROS further activate PCD and UPP, which in turn impairs rubber biosynthesis. To mitigate further injury, rubber trees activate PCD; consequently, latex yield declines or even ceases entirely, ultimately leading to TPD [58]. Interestingly, the transcript abundance of *HbsHSP23.8a* was progressively upregulated along with the onset and progression of TPD in rubber trees (Figure 4). Furthermore, ET, a well-characterized inducer of TPD, markedly activated *HbsHSP23.8a* expression in the present study (Figure 3C). Previous studies have demonstrated that excessive ROS accumulation in laticifers acts as a central driver of TPD occurrence in rubber trees [57,60]. Consistently, the genes involved in ROS scavenging and the target genes of TPD-related microRNAs associated with ROS-scavenging systems exhibit substantial differential expression between TPD-affected and healthy rubber trees [54,55,57,58,59]. Heterologous overexpression of *HbsHSP23.8a* is associated with enhanced salt tolerance in transgenic tobacco, which may be attributed to elevated antioxidant enzyme activities and reduced cellular H_2_O_2_ contents. Integrating these findings with established ROS-TPD regulatory evidence, we propose a testable hypothesis: beyond its conserved function in salt stress tolerance, *HbsHSP23.8a* probably modulates TPD progression in rubber trees via orchestrating endogenous ROS scavenging homeostasis. Therefore, our study reveals a potential functional connection between an sHSP family member and TPD-associated ROS signaling in rubber trees. Despite this putative correlative evidence linking *HbsHSP23.8a* to TPD-related ROS signaling, the direct biological functions of *HbsHSP23.8a* during TPD onset and development require further validation through in planta functional assays.

Yang et al. (2020) previously reported that salinity stress leads to chlorophyll degradation and excessive H_2_O_2_ accumulation in rubber tree seedlings [25]. In the present study, salt treatment significantly upregulated the transcript level of *HbsHSP23.8a* in rubber trees. Consistent with its salt-induced expression pattern and the results from Yang et al. [25], transgenic tobacco plants overexpressing *HbsHSP23.8a* retained higher chlorophyll content and accumulated less H_2_O_2_ than WT plants under salt treatment. Meanwhile, the activities of three key antioxidant enzymes (CAT, SOD, and POD) were substantially elevated in transgenic lines relative to WT plants. These physiological indices exhibited coordinated variation trends, with antioxidant enzyme activities positively correlated with chlorophyll retention and negatively correlated with H_2_O_2_ accumulation. Moreover, *HbsHSP23.8a*-overexpressing tobacco lines displayed superior growth performance, including significantly increased plant height and fresh weight, under salt stress conditions. Notably, the phenotypic and physiological improvements in transgenic tobacco were tightly correlated with the expression of exogenous *HbsHSP23.8a*, but not the endogenous homologous gene (*NbsHSP24.3*), definitively verifying that the enhanced salt tolerance is related to *HbsHSP23.8a* overexpression. Our findings characterize *HbsHSP23.8a* as a novel and functional candidate gene for molecular breeding of salt-tolerant rubber tree germplasm. This study also provides innovative insights into the molecular regulatory mechanism of sHSP genes underlying plant salt tolerance.

## 4. Materials and Methods

### 4.1. Plant Materials and Treatments

Reyan 7-33-97, a high-yielding rubber tree cultivar, was planted at the experimental station in Danzhou, Hainan province, China. These trees were subjected to conventional tapping (half-spiral cut, once every 4 days) with 1.5% ET stimulation for the preceding ten years. Leaves, latex, shoot apices, barks, female and male flowers were separately collected from 21-year-old rubber trees with consistent growth status. In addition, the leaves at five developmental stages including red, pale-green young, light young, mature and senescence periods were individually harvested at the same time. Bark samples were also collected from TPD-affected rubber trees at the initial and advanced stages (IST and AST) and healthy ones (H) according to the criteria of Yue et al. (2024) [59]. Based on the method described by Hao and Wu (2000) [61], seven-year-old untapped Reyan 7-33-97 trees with uniform growth performance were separately treated with 1% MeJA, or 1% ET before the first tapping to harvest corresponding latex samples. The first few drops of latex with debris were discarded. Latex exuded from treated and control rubber trees was separately collected at 0 h, 4 h, 8 h, 24 h, 48h and 72 h by direct dripping into liquid nitrogen within an ice-kettle. Tissue-cultured Reyan 7-33-97 seedlings with uniform growth were subjected to stress treatments, including low temperature (8 °C) and high salt (1 M NaCl); pale-green young leaves were harvested from both control and treated seedlings at four time points (0 h, 3 h, 24 h and 48 h). Throughout all experiments, only rubber trees or seedlings exhibiting consistent morphological and growth performance were selected as experimental materials. For the aforementioned experimental materials, three independent biological replicates were set up, with each biological replicate consisting of the tissues pooled equally from five uniformly grown rubber trees. All the aforementioned collected experimental samples were immediately frozen in liquid nitrogen for total RNA extraction.

### 4.2. Full-Length cDNA and Expression Profiles of HbsHSP23.8a

Total RNA was extracted from all collected samples with the Plant Total RNA Extraction Kit (TIANGEN Biotech, Beijing, China). Residual genomic DNA contamination was removed via digestion with RNase-free DNase I (Vazyme Biotech, Nanjing, China). RNA integrity was verified by 1% agarose gel electrophoresis, while RNA concentration was quantified using a NanoDrop spectrophotometer. Qualified total RNA was reverse-transcribed into first-strand cDNA with HiScript Reverse Transcriptase (Vazyme Biotech, Nanjing, China).

Full-length cDNA of *HbsHSP23.8a* was obtained via rapid amplification of cDNA ends (RACE) combined with Sanger sequencing. Briefly, 5′ and 3′ terminal fragments of *HbsHSP23.8a* were amplified following the manufacturer’s protocol of the HiScript-TS 5′/3′ RACE Kit (Vazyme Biotech, Nanjing, China), using gene-specific primers (GSPs) and nested gene-specific primers (NSPs) listed in Table S1. All RACE PCR products were inserted into the pNC-AEnTopo vector (NC Biotech, Changsha, China) and validated by bidirectional sequencing. The full-length *HbsHSP23.8a* sequence was assembled by aligning the 5′-RACE, 3′-RACE and internal assembled sequence. The complete sequence was further amplified with the full-length primer pair flHbsHSP23.8aF and flHbsHSP23.8aR (Table S1) and verified by sequencing. All PCR procedures were conducted as previously described by Deng et al. (2018) [62].

For quantitative real-time PCR (qRT-PCR), the expression level of *HbsHSP23.8a* was detected using the gene-specific primers qHbsHSP23.8aF and qHbsHSP23.8aR. The rubber tree 18S rRNA gene (GenBank accession no. AB268099) was selected as the internal reference gene for normalization (Table S1). qRT-PCR reactions were performed on a CFX96 Touch Real-Time PCR Detection System (Bio-Rad Laboratories, Hercules, CA, USA) according to Deng et al. (2018) [62]. The relative transcript abundance of *HbsHSP23.8a* was calculated using the 2^−ΔΔCt^ method [63]. All qRT-PCR experiments were performed with three independent biological replicates, and the relative expression profiles were presented as the mean ± standard deviation (SD) of three biological replicates.

### 4.3. Bioinformatic Analyses

The amino acid sequence, theoretical molecular mass, and pI of HbsHSP23.8a were predicted using DNASTAR 7.0 software. In addition, the conserved domains of HbsHSP23.8a were identified via the NCBI Conserved Domain Database (CDD) search tool. Homologous sequences of *HbsHSP23.8a* were retrieved by BLAST analysis against the Nicotiana benthamiana genome database NbenBase (https://nbenthamiana.jp/) (accessed on 29 June 2026). The locus Nbe.v1.1.chr15g31300 was identified as the tobacco ortholog of *HbsHSP23.8a*. Based on its predicted molecular weight, this gene was named *NbsHSP24.3* in this study. Gene-specific primers qNbsHSP24.3F and qNbsHSP24.3R were designed to quantify *NbsHSP24.3* transcript levels using qRT-PCR (Table S1).

### 4.4. Generation of HbsHSP23.8a Overexpression Transgenic Tobacco Plants

To investigate the biological function of *HbsHSP23.8a*, the full-length ORF of *HbsHSP23.8a* was amplified using gene-specific primers OE-HbsHSP23.8aF and OE-HbsHSP23.8aR (Table S1) and inserted into the pCAMBIA1301 expression vector. The recombinant plasmid was introduced into *Nicotiana benthamiana* via *Agrobacterium tumefaciens*-mediated leaf disk transformation, following the protocol described by Rajput et al. (2014) [64].

Transgenic T_0_ plants were initially screened on Murashige and Skoog (MS) medium supplemented with 30 mg/L hygromycin. Positive transformants were further verified by PCR amplification using primers 35SF and HbsHSP23.8aR (Table S1). Seeds harvested from T_0_ plants were collected as the T_1_ generation and subjected to hygromycin resistance segregation analysis. Transgenic lines with an approximate 3:1 resistance segregation ratio, indicating a single-copy transgene insertion, were selected for subsequent assays. Hygromycin-resistant T_1_ plants were self-pollinated to produce T_2_ generation seeds. Segregation analysis was repeated on T_2_ seeds, and homozygous lines (exhibiting 100% hygromycin-resistant seedlings) were selected to harvest T_3_ seeds.

qRT-PCR was performed to detect the transcript levels of *HbsHSP23.8a* and its tobacco ortholog *NbsHSP24.3* in T_3_ homozygous lines. All experiments were conducted with three independent biological replicates, and each replicate consisted of evenly mixed leaf tissues collected from five uniformly grown seedlings. Total RNA was extracted from *N. benthamiana* leaves using Trizol reagent (Beyotime, China). First-strand cDNA synthesis and qRT-PCR were conducted as described previously. The elongation factor 1α (*EF1α*) gene of *N. benthamiana* was used as the internal reference gene for data normalization, and primers NbEF1aF and NbEF1aR (Table S1) were used for EF1α amplification [64]. Based on the results of segregation analysis and expression profile, three independent T_3_ homozygous lines (OE1, OE3, and OE6) were selected for subsequent gene function characterization.

### 4.5. Salt Stress Tolerance Analysis

The seeds of WT and T_3_ homozygous transgenic tobaccos were surface-sterilized and sown on solid MS medium under the following conditions: a day/night temperature regime of 25/23 °C, a photoperiod of 16 h light/8 h dark, and a light intensity of 200 μmol m^−2^ s^−1^. After 7 days, uniformly sized WT and transgenic seedlings were transferred to fresh MS medium supplemented with 0 mM NaCl (control) or 180 mM NaCl for salt stress treatment. Following ten days of continuous cultivation in the growth chamber under identical environmental conditions, seedling plant height and fresh weight were determined. All samples were performed with three biological replicates, and each biological replicate consisted of five individual seedlings with uniform growth performance to minimize random individual differences.

Leaves from the WT and transgenic line seedlings were separately harvested to determine five physiological parameters. The H_2_O_2_ content was extracted with 5% (*w*/*v*) trichloroacetic acid (TCA) and quantified according to the method described by Patterson et al. (1984) [65]. The chlorophyll was extracted using 95% ethanol and measured by the spectrophotometer according to the method [66]. The activities of three major antioxidant enzymes including CAT, SOD, and POD were measured using commercial assay kits (Solarbio, China) in accordance with the manufacturer’s instructions. All physiological measurements were performed with three independent biological replicates. Each biological replicate consisted of equivalently pooled leaf tissues from five uniformly grown seedlings to reduce individual plant variation and ensure sample consistency.

### 4.6. Statistical Analysis

All data are presented as the mean ± standard deviation (SD) of three independent biological replicates. Statistical analyses and data visualization were implemented with custom Python scripts relying on NumPy, SciPy, pandas, and Matplotlib. Duncan’s multiple range test (DMRT) was integrated into this Python workflow for post hoc multiple comparisons. Residual normality and homogeneity of variance were checked prior to ANOVA to validate model assumptions.

For single-factor experimental designs (e.g., comparisons across tissues, leaf developmental stages, sampling time points, or genotypes under one fixed condition), each variable was subjected to one-way analysis of variance (ANOVA), followed by DMRT for post hoc multiple comparisons. Different lowercase letters denote distinct statistical groups at *p* < 0.05; groups with non-overlapping letters differ significantly, whereas groups sharing at least one common letter show no significant difference.

For physiological parameters determined under 0 and 180 mM NaCl, two-way ANOVA was separately carried out for each parameter to assess the main effects of genotype, salt treatment, and their genotype × salt treatment interaction. The *p*-value of the genotype × salt treatment interaction is presented in the corresponding panel of each figure (*** *p* < 0.001). Upon detection of a significant genotype × salt treatment interaction, follow-up comparisons were performed to dissect simple effects. Genotypic comparisons within each salt concentration were conducted using DMRT based on the pooled residual mean square error (MSE) derived from the two-way ANOVA model. Lowercase and uppercase letters represent genotype groupings under 0 mM and 180 mM NaCl, respectively (*p* < 0.05). Within each genotype, two-sided t-tests with Sidak correction for multiple comparisons (using the same pooled residual MSE) were used to test differences between 0 and 180 mM NaCl treatments. Asterisks mark significant differences between the two salt levels within a given genotype according to Sidak-adjusted *p* values (* *p* < 0.05, ** *p* < 0.01, and *** *p* < 0.001).

## 5. Conclusions

In the present study, *HbsHSP23.8a* was successfully cloned and systematically characterized from rubber trees. Structural analysis revealed that the HbsHSP23.8a protein contained a targeting peptide, N-terminal domain, ACD, and C-terminal extensions, with a predicted dimer interface in the ACD and conserved amino acid sequences in the N-terminal domain. *HbsHSP23.8a* differently expressed in multiple tissues and leaf developmental stages. In addition to its association with TPD, *HbsHSP23.8a* was also responsive to low temperature, high salinity, MeJA, and ET treatments. Under salt stress, tobacco plants overexpressing *HbsHSP23.8a* exhibited greater growth performance compared with WT plants. Moreover, *HbsHSP23.8a* overexpression markedly enhanced the activities of antioxidant enzymes, alleviated H_2_O_2_ accumulation and chlorophyll degradation in salt-stressed tobacco plants. Given the intricate molecular mechanisms underlying the salt tolerance conferred by *HbsHSP23.8a* and its potential application, future investigations could focus on characterizing the subcellular localization of HbsHSP23.8a, screening its interacting proteins, identifying up- and down-stream genes, unraveling associated signaling cascades, analyzing antioxidant regulatory systems, and generating gene knockout mutants via loss-of-function strategies to systematically elucidate the molecular basis by which *HbsHSP23.8a* boosts plant stress tolerance.

## Data Availability

The original contributions presented in the study are included in the article/Supplementary Material. Further inquiries can be directed to the corresponding author.

## Supplementary Materials

The following supporting information can be downloaded at: https://www.mdpi.com/article/10.3390/plants15213318/s1, Table S1: the primer sequences used for the experiment in this study.
