# Supplementary material for: Molecular Cloning, Expression Profiles, Characterization and Function of a Novel Small Heat Shock Protein (HbsHSP23.8a) in Hevea brasiliensis
Source: Plants (Basel). 2026 Oct 26;15(21):3318. doi: 10.3390/plants15213318 (PMC13765897; doi:10.3390/plants15213318)
Supplement: Supplementary file 1 [file plants-15-03318-s001.zip › plants-4604087-supplementary.pdf]

Table S1 The primers used in this study.

Gene name	Primer sequences
5′-RACE primer 1	5′-GCAGATAGGTAGGGACAAGG-3′
5′-RACE primer 2	5′-GTGAGGAAGCCATTGCTTGGAG-3′
3′–RACE primer 1	5′-GGTACACCAGTAGGATTGATC-3′
3′–RACE primer 2	5′-GGTGTGTTGAAGGTTGTTGTG-3′
flHbsHSP23.8aF	5′-GCTCGATGAGCTGTCTTTCG-3′
flHbsHSP23.8aR	5′-CCAGTTACGTTACTCTTAATCG-3′
qHbsHSP23.8aF	5′-CTTCTCAGACGTGGTGGATC-3′
qHbsHSP23.8aR	5′-GGTCTCACGAACGTCCCAG-3′
NbEF1αF	5′-TTGCTTGCTTTCACCCTTGG-3′
NbEF1αR	5′-TCGAAACCAGAGA TGGGGAC-3′
OE-HbsHSP23.8aF	5′-CATCCATGGCTTCCTCACTGGCTC-3′
OE-HbsHSP23.8aR	5′-TAAGGTGACCTTACTCGACCTTGACATGGAA-3′
35SF	5′-ACGCACAATCCCACTATCCTTC-3′
HbsHSP23.8aR	5′-GGAGGTAGAGAGGAAAGGG-3′
qNbsHSP24.3F	5′-GTCAACCCTGTTCGCTCTG-3′
qNbsHSP24.3R	5′-GGTCAACATCGATGGTGCG-3′
Hb18SrRNAF	5′-GCTCGAAGACGATCAGATACC-3′
Hb18SrRNAR	5′-TTCAGCCTTGCGACCATAC-3′
